# HGSNAT alleviates oxygen-glucose deprivation- induced endothelial injury by suppressing ER stress: implications for Moyamoya disease

**DOI:** 10.3389/fnmol.2026.1839595

**Published:** 2026-08-10

**Authors:** Zhonghui Wen, Bin Hu, Hai Wang, Ziyu Chen, Sen Yang, Fuqing Zhang, Zhiwei Tang

**Affiliations:** 1 Department of Neurosurgery, The First Affiliated Hospital of Kunming Medical University, Kunming, Yunnan, China; 2 Department of Neurosurgery, People’s Hospital of Pu’er City, Pu’er, Yunnan, China; 3 Department of Neurology, People’s Hospital of Pu’er City, Pu’er, Yunnan, China; 4 Department of Nephrology, People’s Hospital of Pu’er City, Pu’er, Yunnan, China

**Keywords:** endoplasmic reticulum stress, endothelial injury, GPX8, HGSNAT, Moyamoya disease, oxygen-glucose deprivation

## Abstract

**Objectives:**

Moyamoya disease (MMD) is a progressive cerebrovascular disorder characterized by chronic cerebral hypoperfusion and ischemic microenvironments that critically impact brain microvascular endothelial cells. Oxygen-glucose deprivation (OGD) models have been extensively employed in MMD research to recapitulate the ischemic conditions relevant to MMD pathophysiology. This study aims to explore the functional involvement and molecular basis of HGSNAT in human brain microvascular endothelial cells (HBMECs) following OGD-induced injury, to establish a theoretical foundation for elucidating the pathogenesis of vascular endothelial injury associated with MMD.

**Methods:**

An *in vitro* model of hypoxic-ischemic injury was established by subjecting HBMECs to OGD. After HGSNAT overexpression, endoplasmic reticulum stress (ERS) activator Tunicamycin treatment, and GPX8 silencing, cell viability, angiogenic capacity, and migratory ability were evaluated using the CCK-8 assay, tube formation assay, and scratch wound healing assay, respectively. Enzyme-linked immunosorbent assay (ELISA) was employed to evaluate the secretion of inflammatory cytokines, while intracytoplasmic reactive oxygen species (ROS) levels were examined using DCFH-DA staining. Additionally, Western blotting was performed to assess the abundance of factors linked to the ERS cascade.

**Results:**

OGD exposure significantly downregulated HGSNAT mRNA expression and enzymatic activity. HGSNAT overexpression markedly enhanced the viability of OGD-treated HBMECs and improved angiogenic and migratory capacities, while reducing the secretion of interleukin-1β (IL-1β), interleukin-6 (IL-6), and tumor necrosis factor-α (TNF-α). Functional studies uncovered that HGSNAT overexpression reduced ROS accumulation and suppressed activation of the PERK-eIF2α-ATF4 cascade; these effects were abrogated by Tunicamycin treatment. Furthermore, the study found that GPX8 knockout could weaken the protective effect mediated by HGSNAT, resulting in decreased cell survival, impaired angiogenesis and migratory ability, elevated inflammatory levels, and reactivation of the ERS pathway. This indicates that the efficacy of HGSNAT required for maintaining endothelial homeostasis depends on the function of GPX8.

**Conclusion:**

Overexpression of HGSNAT is associated with maintaining or enhancing GPX8 levels under OGD stress, thereby protecting HBMECs from OGD-induced endoplasmic reticulum stress and endothelial damage. GPX8 knockdown partially abolished these protective effects, suggesting that GPX8 contributes to HGSNAT-mediated inhibition of the PERK–eIF2α–ATF4 ER stress pathway, oxidative stress, and inflammation.

## 1 Introduction

Moyamoya disease (MMD) is a rare and progressive intracranial vascular occlusive syndrome (He et al., 2026), characterized by progressive stenosis of the terminal internal carotid arteries and their principal divisions, along with the formation of abnormal compensatory vessels (Cheng et al., 2025).

Although the underlying etiology of MMD remains incompletely understood, vascular endothelial cell dysfunction is widely recognized as a central mechanism in its pathogenesis (Han et al., 2025; Ma et al., 2023). As key structural and functional elements of the blood-brain barrier (BBB), brain microvascular endothelial cells (BMECs) are essential components (Galpayage Dona et al., 2023); consequently, their injury and dysfunction result in BBB disruption and cerebral hemodynamic disturbances (Tang et al., 2025). These pathological changes trigger secondary ischemic injury, thereby exacerbating disease progression (Zhang and Fan, 2025). Accumulating evidence indicates that, in addition to chronic cerebral hypoperfusion and aberrant vascular remodeling, inflammatory processes are increasingly recognized as important contributors to the pathogenesis and progression of MMD. Although MMD has traditionally been considered a non-inflammatory cerebrovascular disorder, growing experimental and clinical studies suggest that sterile inflammation may participate in endothelial dysfunction, blood-brain barrier disruption, and pathological vascular remodeling (Abhinav et al., 2024; Mikami et al., 2019). Therefore, elucidating the key molecular drivers underlying BMEC dysfunction is imperative for uncovering the mechanisms of MMD and identifying novel therapeutic targets.

Endoplasmic reticulum stress (ERS) stems from the increase in misfolded proteins and can induce apoptosis and inflammation upon sustained and excessive activation, thereby contributing to vascular endothelial injury (Cao et al., 2025; Musielak et al., 2024; Zhang et al., 2026). Emerging evidence has shown significantly abnormal ERS activation within vascular tissues of MMD patients, underscoring its pathogenic significance in vascular endothelial dysfunction. These observations imply that targeting aberrant ERS might constitute an effective therapeutic intervention to mitigate vascular injury in MMD (Han et al., 2021). Heparan-α-glucosaminide N-acetyltransferase (HGSNAT) is a transmembrane enzyme localized to the lysosomal membrane, functioning as an acetyltransferase (Pan et al., 2022). Of note, HGSNAT deficiency induces protein misfolding and subsequent ER retention, pointing to a mechanistic connection between HGSNAT dysfunction and ERS (Pshezhetsky, 2015). This raises the possibility that HGSNAT may contribute to vascular pathology via ERS modulation. Glutathione peroxidase 8 (GPX8) is an ER-resident antioxidant enzyme that maintains ER redox homeostasis and negatively regulates ERS. Downregulation of HGSNAT may initiate a pathological cascade characterized by reduced GPX8, aberrant ERS activation, and subsequent endothelial injury, ultimately contributing to MMD pathogenesis. We therefore hypothesize that HGSNAT preserves brain microvascular endothelial cell homeostasis by upregulating GPX8, thereby restraining excessive ERS. However, whether HGSNAT influences MMD-associated vascular endothelial injury via the GPX8/ERS axis—particularly through suppression of the PERK-eIF2α-ATF4 pathway—has yet to be reported, and the exact molecular mechanism has yet to be clarified.

The OGD model has been extensively validated as an *in vitro* platform for investigating MMD-associated endothelial pathophysiology. Recent studies have demonstrated that OGD-exposed brain microvascular endothelial cells recapitulate key features of MMD-related ischemic injury, including impaired angiogenesis and increased inflammatory responses. For instance, Shin et al. employed OGD-treated endothelial cells to investigate the role of RNF213 variants in MMD pathogenesis (Shin et al., 2024). Similarly, a recent study utilized OGD-treated brain microvascular endothelial cells to model MMD-associated ischemic injury and evaluate therapeutic agents (Gao et al., 2026). Thus, while OGD does not fully encompass the complex etiology of MMD, it provides a well-established and reproducible system to investigate endothelial dysfunction in the context of chronic cerebral hypoperfusion—a hallmark of MMD pathology. In the present study, an OGD model was established using human brain microvascular endothelial cells (HBMECs) to investigate how HGSNAT regulates ER stress through the PERK-eIF2α-ATF4 cascade and to validate the critical involvement of GPX8 in this signaling axis. Findings from this investigation offer novel insights into the regulatory processes underlying vascular endothelial injury in MMD.

## 2 Materials and methods

### 2.1 Cell culture

Human brain microvascular endothelial cells were obtained from the Cell Bank of the Chinese Academy of Sciences in Shanghai, China. Cells were cultured in SILAC Advanced DMEM and F-12 Flex medium (Gibco™, USA) containing 10% premium fetal bovine serum (Gibco™, Australia) and 1% penicillin-streptomycin (Gibco™, USA) and incubated under appropriate conditions in a CO_2_ incubator Heracell™ VIOS 160i (Thermo Scientific™, USA).

### 2.2 OGD model establishment and treatment

To mimic the MMD-related hypoxic-ischemic microenvironment, HBMECs at 70%–80% confluence were incubated in glucose-free DMEM under 1% O_2_, 5% CO_2_, and 94% N_2_ at 37 °C for 6 h to induce the OGD model. The selection of 1% O_2_ (severe hypoxia) instead of 3% O_2_ (moderate or physiological hypoxia) was based on previous literature establishing that severe hypoxia is required to reliably trigger notable cellular dysfunction, reactive oxygen species (ROS) accumulation, and robust ERS activation in HBMECs *in vitro* (Yang et al., 2021; Zhang et al., 2016). Conversely, a milder hypoxia of 3% O_2_ is typically tolerated by endothelial cells as a physiological adaptation, which fails to induce the reproducible ischemic-like endothelial injury necessary to model Moyamoya disease pathological events (Warpsinski et al., 2020). Control cells were maintained in complete medium under normoxia (21% O_2_, 5% CO_2_). To investigate the link between ER stress and HGSNAT, cells were pretreated with tunicamycin (Selleck, USA) for 2 h before OGD.

### 2.3 Cell transfection

For transfection, HBMECs were seeded in 6-well plates at an initial density of 2 × 10^5^ cells/well and cultured until they reached 70%–80% confluence. Cells were transiently transfected with HGSNAT overexpression plasmids (oe-HGSNAT or negative control oe-NC, 2.5 μg/well) or GPX8-targeting siRNA (si-GPX8 or negative control si-NC, 50 nM) using Lipofectamine 3,000 transfection reagent (Invitrogen, USA) according to the manufacturer’s protocols. The transfection efficiency was evaluated after 48 h and reached approximately 75%–80% based on fluorescence microscopy of green fluorescent protein (GFP)-labeled control plasmids. The transfection efficiency was further verified quantitatively by RT-qPCR and Western blotting before subsequent OGD treatment, and functional assays were conducted.

### 2.4 RNA extraction and rT-qPCR analysis

TRIzol reagent was applied for total RNA extraction from HBMECs, which was then quantified and assessed for purity. After reverse transcription, qPCR was conducted with SYBR Green. The primer sequences used were as follows: HGSNAT forward, 5′-CCAGACTGGGTCACCAAACA-3′, reverse, 5′-AGTA TTTTTCCTGCTTCCAGGC-3′; GAPDH forward, 5′-TGTTTC CTCGTCCCGTAG-3′, reverse, 5′-CAATCTCCACTTTGCCA CT-3′.

### 2.5 Enzyme-linked immunosorbent assay (ELISA)

To determine the concentrations of inflammatory mediators (TNF-α, IL-6, and IL-1β), ELISA reagent kits (Beyotime, China) and high-binding microplates (ESP-96-D, Sevier Biologics, China) were employed according to the manufacturer’s instructions. The absorbance was measured at the specified wavelength using a microplate reader, and the levels of these cytokines were calculated based on the corresponding standard curves.

### 2.6 HGSNAT enzymatic activity assay

The enzymatic activity of HGSNAT in HBMECs was measured using a fluorometric assay with the synthetic substrate 4-methylumbelliferyl-β-D-glucosaminide (Sigma-Aldrich, USA) as previously described (Pan et al., 2022; Pshezhetsky, 2015). Briefly, cells were harvested and lysed by three freeze-thaw cycles in distilled water. The protein concentration of the supernatant was determined using the BCA protein assay kit. Then, 20 μL of cell lysate was incubated with 20 μL of 3 mM 4-methylumbelliferyl-β-D-glucosaminide and 10 μL of 10 mM acetyl-CoA in 0.5 M sodium citrate buffer (pH 5.5) at 37 °C for 17 h. The reaction was terminated by adding 1 mL of 0.5 M glycine-NaOH buffer (pH 10.5). Fluorescence was measured using a microplate reader (excitation 365 nm, emission 450 nm). HGSNAT activity was calculated as nmol of 4-methylumbelliferone released per mg of protein per hour (units/mg protein).

### 2.7 Cell viability assay

Cells were seeded in 96-well plates, treated, incubated with CCK-8 solution, and cell viability was measured by absorbance.

### 2.8 Wound healing assay

Human brain microvascular endothelial cells were seeded in 6-well plates and cultured until 100% confluence. A sterile 200 μL pipette tip was used to create a linear scratch in the cell monolayer. After being washed three times with PBS to remove cellular debris, the cells were incubated in serum-free medium. Representative images of the scratched area were captured at 0 and 48 h using an inverted microscope (Olympus, Japan). The migration rate was calculated using ImageJ software.

### 2.9 Tube formation assay

Matrigel (Corning, USA) was first liquefied at 4 °C and then dispensed into 96-well plates at 50 μL per well, after which polymerization was conducted at 37 °C for 30 min. HBMECs were seeded in 100 μL complete medium. After 6 h, tube formation was imaged and quantified by counting tubules and branch points.

### 2.10 Measurement of ROS

2’,7’-Dichlorodihydrofluorescein diacetate (DCFH-DA) staining was employed to assess intracellular ROS levels. Cells following treatment were exposed to 10 μmol/L DCFH-DA for 20 min. ROS was observed by confocal laser microscopy, and fluorescence intensity was quantified with ImageJ.

### 2.11 Western blotting

Western blotting was done following previously published protocols (Guo et al., 2024). The following primary antibodies were employed: rabbit anti-HGSNAT (Abcam, ab192398, 1:1000), rabbit anti-GRP78 (Abcam, ab21685, 1:1000), rabbit anti-ATF4 (Abcam, ab186730, 1:1000), rabbit anti-PERK (Abcam, ab229912, 1:1000), rabbit anti-p-PERK (Abcam, ab192591, 1:1000), mouse anti-eIF2α (Abcam, ab169519, 1:1000), rabbit anti-p-eIF2α (Abcam, ab32157, 1:1000), rabbit anti-GPX8 (Abcam, ab183665, 1:1000), mouse anti-GAPDH (Abcam, ab8226, 1:5000), and mouse anti-β-actin (Abcam, ab8227, 1:5000). The secondary antibodies included HRP-conjugated goat anti-rabbit IgG (Abcam, ab6721, 1:5000) and HRP-conjugated goat anti-mouse IgG (Abcam, ab6789, 1:5000). Membrane-bound proteins were detected by ECL and quantified with ImageJ.

### 2.12 Statistical analysis

Statistical analyses were performed using SPSS version 26.0. Data visualization and graph generation were performed using GraphPad Prism 9.0. Data are presented as the mean ± SD from three independent biological replicates (*n* = 3 per group), with individual data points superimposed on the bars to visualize the distribution and reproducibility of each independent experiment. An independent Student’s *t*-test was used for comparisons between two groups, whereas one-way ANOVA followed by Tukey’s *post-hoc* test was used for comparisons among multiple groups. A two-sided *P* < 0.05 was considered statistically significant.

## 3 Results

### 3.1 Downregulation of HGSNAT under MMD-mimicking conditions and its protective role in endothelial cells

**FIGURE 1:**
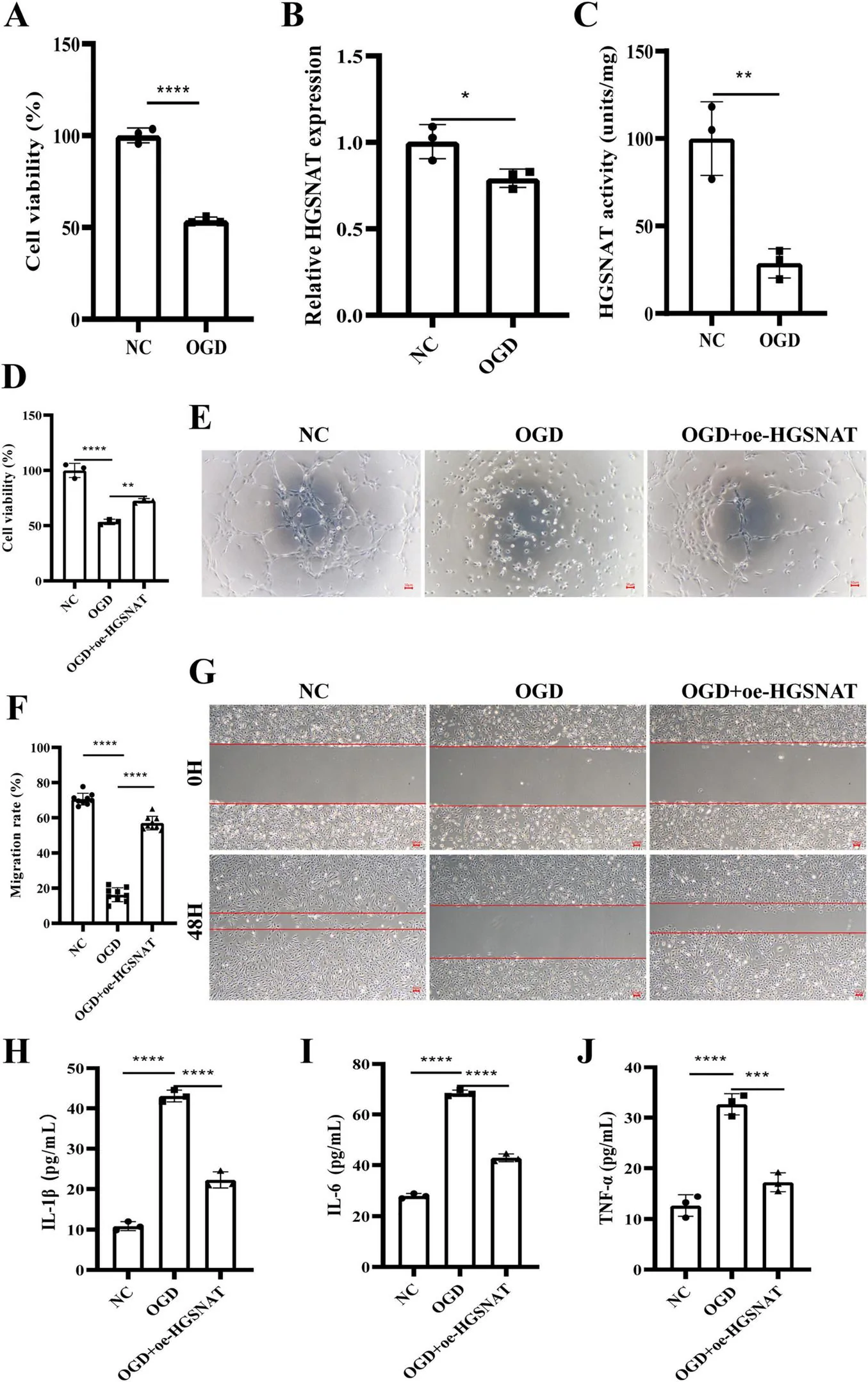
Heparan-α-glucosaminide N-acetyltransferase (HGSNAT) expression is downregulated under oxygen-glucose deprivation (OGD) conditions, and its overexpression alleviates OGD-induced cell damage, endothelial dysfunction, and inflammation in human brain microvascular endothelial cells (HBMECs). **(A)** Cell viability was assessed by CCK-8 assay under NC and OGD conditions. **(B,C)** Relative mRNA expression and enzymatic activity of HGSNAT under NC and OGD conditions. **(D)** Cell viability of HBMECs under NC, OGD, and OGD+oe-HGSNAT conditions. **(E)** Angiogenic capacity evaluated by tube formation assay (scale bar = 50 μm). **(F,G)** Migratory potential and rates were determined by scratch wound healing assay at 0 and 48 h (scale bar = 100 μm). **(H–J)** ELISA measured concentrations of inflammatory cytokines (IL-1β, IL-6, and TNF-α). Data are presented as mean ± SD (*n* = 3). *P < 0.05, **P < 0.01, ***P < 0.001, ****P < 0.0001.

Moyamoya disease typically presents with gradual stenosis of the terminal internal carotid arteries, which leads to sustained cerebral hypoperfusion and creates an ischemic microenvironment affecting BMECs. OGD-treated HBMECs model MMD-associated endothelial ischemic injury and serve as a classic *in vitro* platform for studying MMD vascular pathology (Li et al., 2024). OGD significantly reduced HBMEC viability versus NC controls (CCK-8, *P* < 0.0001, Figure 1A). To elucidate the expression dynamics of HGSNAT under these pathological conditions, we assessed its mRNA levels and enzymatic activity. The results indicated that OGD exposure markedly downregulated both HGSNAT mRNA expression (*P* < 0.05, Figure 1B) and enzymatic activity (*P* < 0.01, Figure 1C), suggesting a potential association between HGSNAT deficiency and OGD-induced cellular injury.

To systematically clarify the dynamic pathological responses of HGSNAT and GPX8 during ischemic-hypoxic progression, we performed a time-course analysis (0, 3, 6, 9, and 12 h) in OGD-treated HBMECs. As shown in Supplementary Figure 1, OGD exposure induced a progressive, time-dependent decline in cell viability (Supplementary Figure 1A), which was accompanied by a continuous downregulation of HGSNAT mRNA expression (Supplementary Figure 1B) and enzymatic activity (Supplementary Figure 1C). Conversely, GPX8 protein levels exhibited a significant, time-dependent upregulation during OGD exposure (Supplementary Figures 1D, E), suggesting an adaptive stress-responsive mechanism of GPX8 under hypoxic-ischemic stress.

To additionally elucidate the involvement of HGSNAT within ischemic damage associated with MMD, an overexpression vector targeting HGSNAT was introduced into HBMECs, and successful transfection was confirmed (Supplementary Figures 2A, B). Functional assays revealed that HGSNAT overexpression significantly rescued the OGD-induced decline in cell viability (*P* < 0.01, Figure 1D). Further phenotypic analyses demonstrated that upregulation of HGSNAT not only enhanced angiogenic capacity (Figure 1E) and migratory potential (*P* < 0.0001, Figures 1F, G) but also markedly attenuated the OGD-triggered inflammatory response. This anti-inflammatory effect was confirmed by reduced IL-1β, IL-6, and TNF-α levels in cell culture supernatants (Figures 1H–J; *P* < 0.0001 for IL-1β and IL-6, *P* < 0.001 for TNF-α). Together, these results indicate that HGSNAT exerts a critical protective function against hypoxic-ischemic damage to endothelial cells, thereby offering novel experimental insights into the molecular mechanisms driving vascular endothelial dysfunction in MMD.

### 3.2 HGSNAT attenuates OGD-induced oxidative stress and ER stress via inhibition of the PERK-eIF2α-ATF4 pathway

**FIGURE 2:**
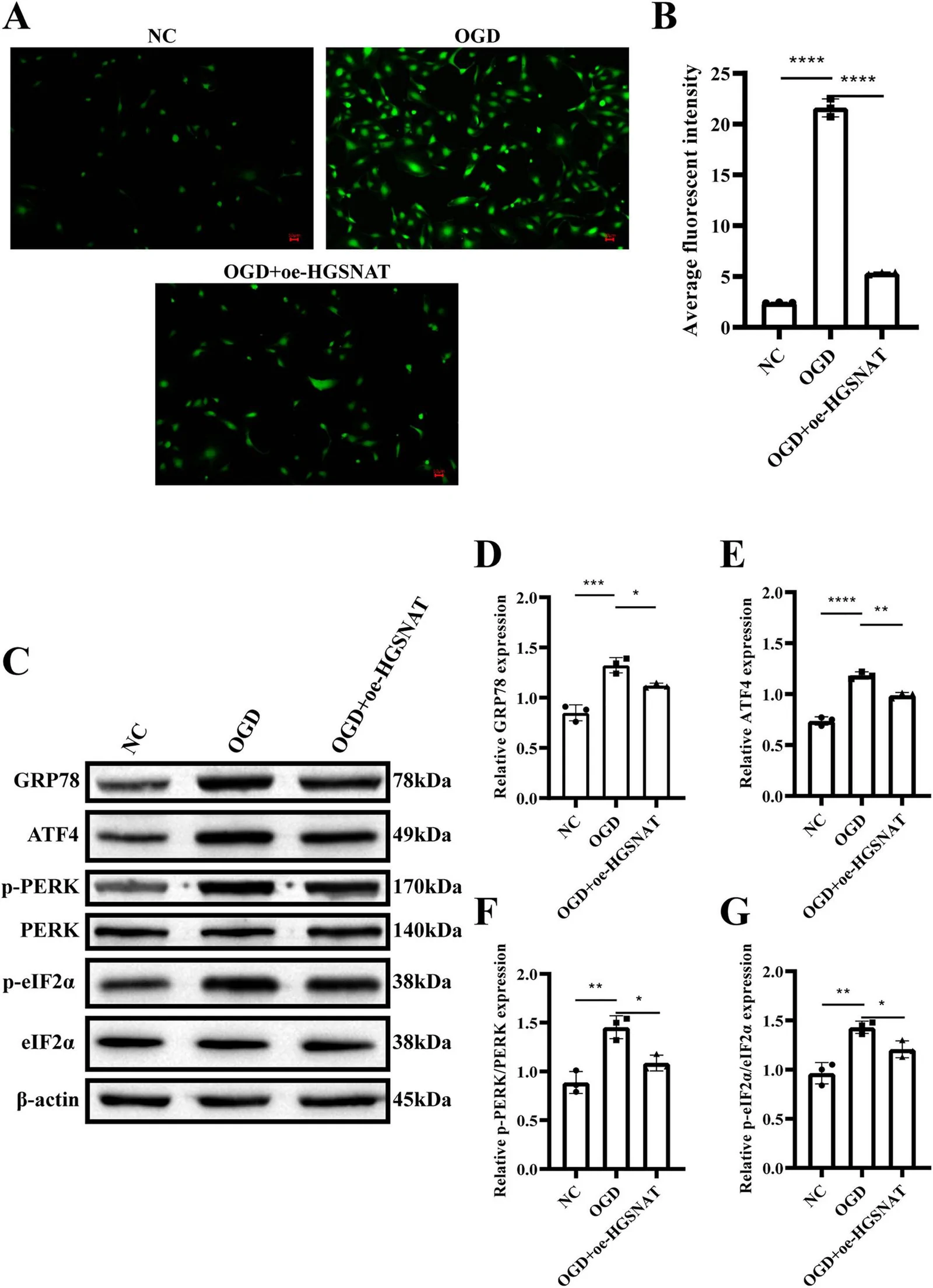
Heparan-α-glucosaminide N-acetyltransferase (HGSNAT) overexpression restrains oxygen-glucose deprivation (OGD)-induced reactive oxygen species (ROS) accumulation and PERK-eIF2α-ATF4 ER stress pathway activation in human brain microvascular endothelial cells (HBMECs). **(A,B)** Representative microphotographs and quantitative relative fluorescence intensity analysis of intracellular ROS levels measured via 2’,7’-Dichlorodihydrofluorescein diacetate (DCFH-DA) staining (scale bar = 50 μm). **(C)** Representative Western blot bands of GRP78, ATF4, p-PERK, PERK, p-eIF2α, and eIF2α proteins, with β-actin serving as the internal loading control. **(D–G)** Statistical quantification of relative protein levels for GRP78, ATF4, p-PERK/PERK, and p-eIF2α/eIF2α. Data are presented as mean ± SD (*n* = 3). *P < 0.05, **P < 0.01, ***P < 0.001, ****P < 0.0001.

Oxidative stress serves as a critical pathological hallmark of MMD (Han et al., 2024). To investigate whether HGSNAT exerts a protective effect by modulating oxidative stress, intracellular ROS levels were assessed using immunofluorescence staining (Figure 2A). Relative to the control condition, the OGD-exposed cells exhibited a marked rise in ROS fluorescence intensity (*P* < 0.0001, Figure 2B), suggesting that exposure to OGD triggered a pronounced oxidative stress response. Conversely, HGSNAT overexpression significantly attenuated ROS accumulation (*P* < 0.0001, Figure 2B), suggesting that HGSNAT effectively mitigates OGD-induced oxidative stress. The observed reduction in ROS accumulation and suppression of ERS markers following HGSNAT overexpression are cell-autonomous effects, as they were consistently observed in HBMECs following direct genetic manipulation of HGSNAT without exogenous factors or co-culture conditions.

Considering the well-recognized interplay between ER stress and oxidative stress under ischemic conditions, together with the fact that the PERK–eIF2α–ATF4 axis serves as a central signaling cascade driving ER stress progression, we next examined the activation of ER stress-related markers along with components within the PERK–eIF2α–ATF4 cascade using Western blotting (Figure 2C). Following OGD exposure, the protein abundance of the ER stress-related marker GRP78 (*P* < 0.001, Figure 2D) and ATF4 (*P* < 0.0001, Figure 2E), together with the p-PERK/PERK ratio (*P* < 0.01, Figure 2F) and p-eIF2α/eIF2α ratio (*P* < 0.01, Figure 2G), were markedly increased, suggesting engagement of the PERK–eIF2α–ATF4 cascade under OGD conditions. Notably, in the HGSNAT-overexpressing group, the protein abundance of GRP78 and ATF4, together with the levels of PERK and eIF2α, was markedly reduced compared with those in the OGD condition (*P* < 0.05, Figures 2C–G).

### 3.3 HGSNAT-mediated endothelial protection is reversed by ER stress activation

**FIGURE 3:**
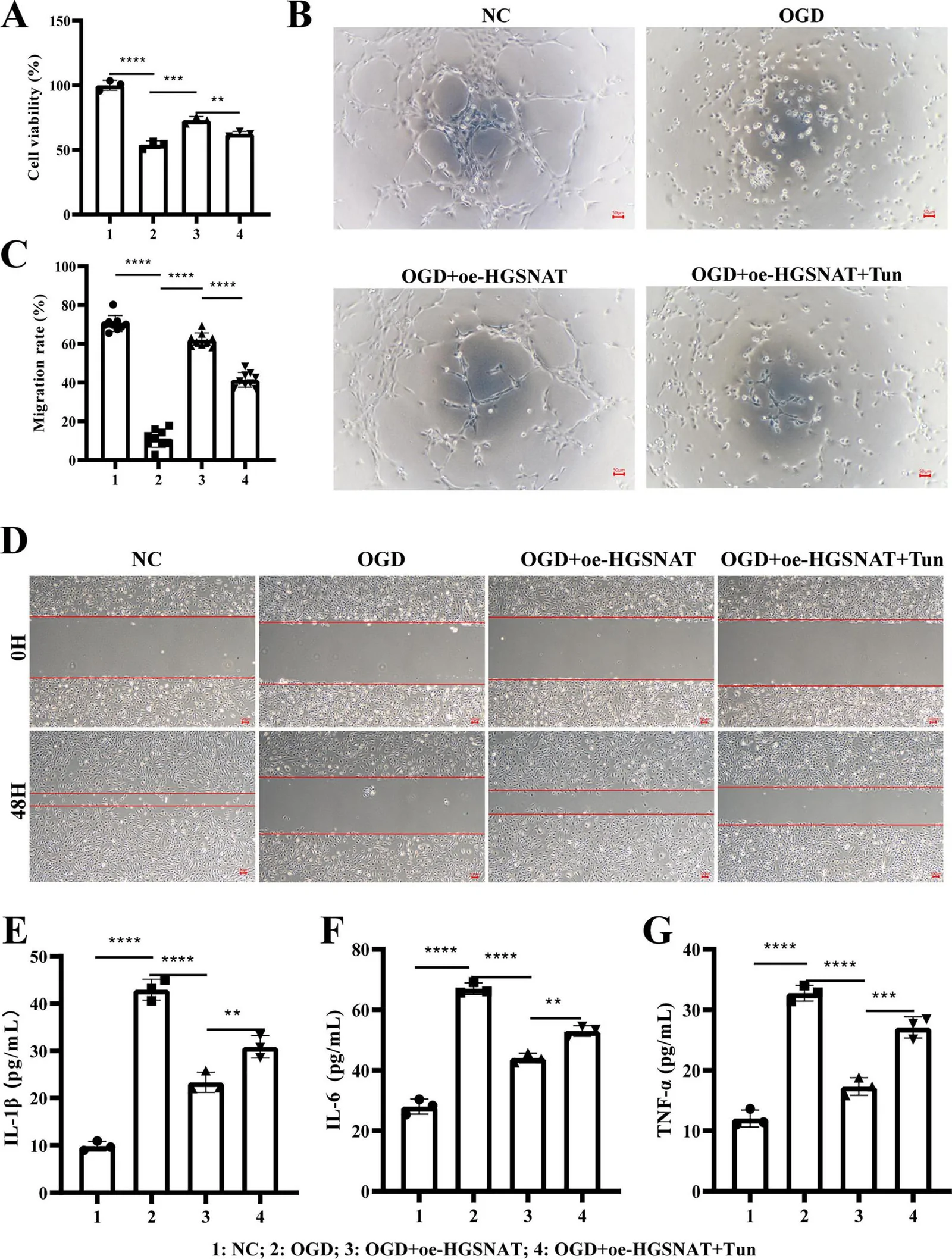
Tunicamycin-mediated ER stress activation counteracts the protective effects of heparan-α-glucosaminide N-acetyltransferase (HGSNAT) overexpression on human brain microvascular endothelial cells (HBMECs) under oxygen-glucose deprivation (OGD) conditions. **(A)** CCK-8 cell viability analysis. **(B)** Representative images of the tube formation assay evaluating angiogenesis (scale bar = 50 μm). **(C,D)** Representative scratch wound healing images and corresponding statistical migration rates of the scratch wound healing assay at 0 and 48 h (scale bar = 100 μm). **(E–G)** Enzyme-linked immunosorbent assay (ELISA) quantification of cellular interleukin-1β (IL-1β), interleukin-6 (IL-6), and tumor necrosis factor-α (TNF-α) inflammatory cytokine secretion. (Groups: 1: NC; 2: OGD; 3: OGD+oe-HGSNAT; 4: OGD+oe-HGSNAT+Tun). Data are presented as mean ± SD (*n* = 3). **P < 0.01, ***P < 0.001, ****P < 0.0001.

To elucidate whether HGSNAT exerts its endothelial protective effects by inhibiting ERS, the ERS activator Tunicamycin (Tun) was administered to OGD-treated HBMECs overexpressing HGSNAT. According to CCK-8 assay results, cell survival was reduced in the Tun-treated group relative to the HGSNAT-overexpressing condition (*P* < 0.01, Figure 3A), indicating that ERS activation partially abrogates the pro-survival benefits conferred by HGSNAT overexpression. Furthermore, tube formation (Figure 3B) and scratch wound healing assays (Figures 3C, D) demonstrated that Tun treatment markedly impaired the angiogenic and migratory capacities enhanced by HGSNAT overexpression, as evidenced by a marked decrease in both tube formation and cell migration (*P* < 0.0001, Figures 3C, D). Additionally, ELISA results revealed that the levels of inflammatory mediators, namely IL-1β (*P* < 0.01, Figure 3E), IL-6 (*P* < 0.01, Figure 3F), and TNF-α (*P* < 0.001, Figure 3G), were significantly higher in the Tun-treated group compared with the HGSNAT-overexpressing condition. These findings indicate that activation of ER stress reverses the inflammatory response induced by OGD and diminishes the anti-inflammatory effect conferred by HGSNAT overexpression.

### 3.4 ER stress activation reverses HGSNAT-mediated suppression of oxidative stress and the PERK-eIF2α-ATF4 axis under OGD

**FIGURE 4:**
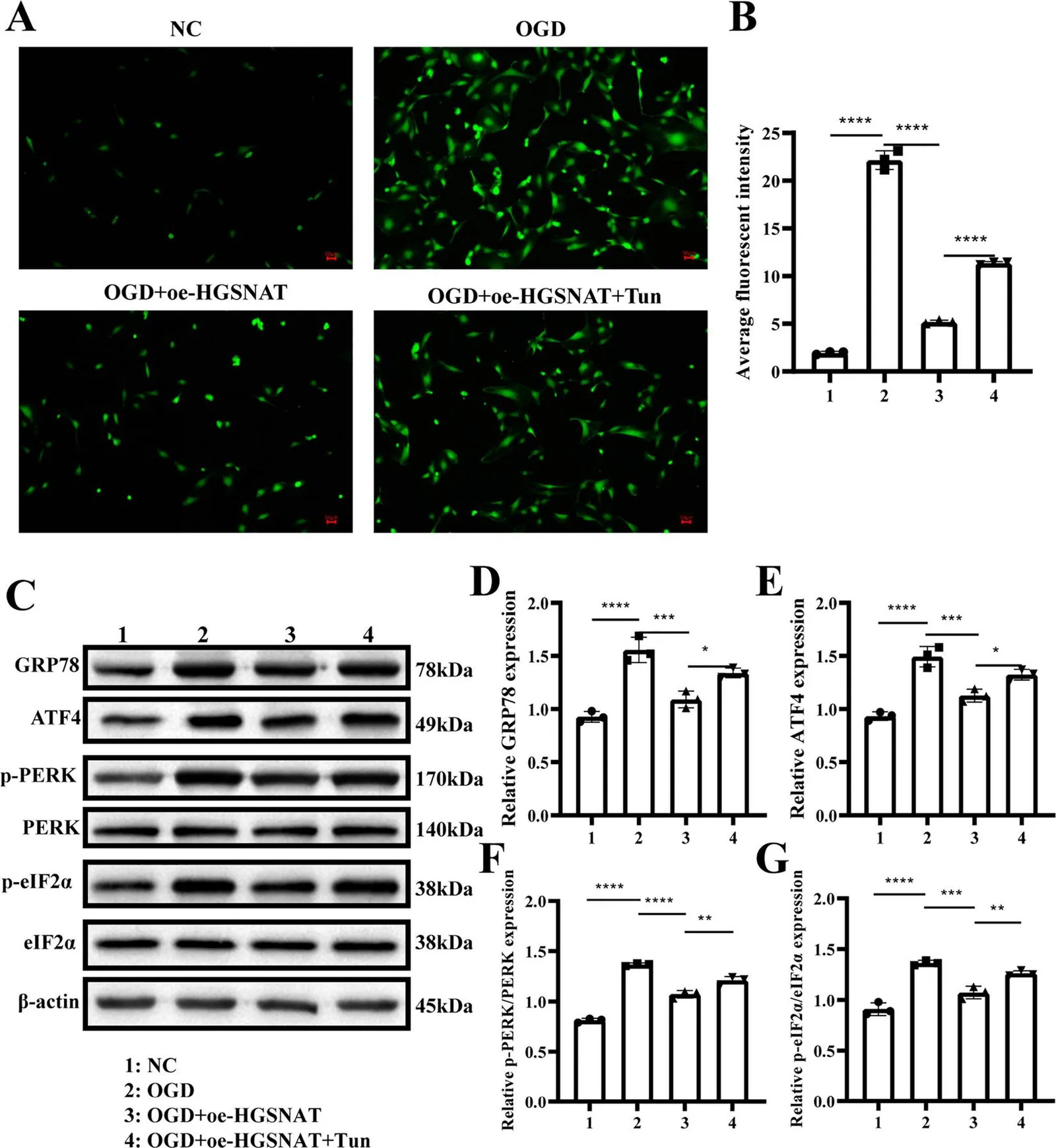
Tunicamycin treatment abolishes the suppressive effect of heparan-α-glucosaminide N-acetyltransferase (HGSNAT) on reactive oxygen species (ROS) and the PERK-eIF2α-ATF4 cascade under oxygen-glucose deprivation (OGD) conditions. **(A,B)** 2’,7’-Dichlorodihydrofluorescein diacetate (DCFH-DA) fluorescent images and calculated fluorescent intensity of cellular ROS production (scale bar = 50 μm). **(C)** Representative Western blot tracks for GRP78, ATF4, p-PERK, PERK, p-eIF2α, and eIF2α. **(D–G)** Densitometric quantification of relative GRP78 and ATF4 levels, along with p-PERK/PERK and p-eIF2α/eIF2α activation ratios. (Groups: 1: NC; 2: OGD; 3: OGD+oe-HGSNAT; 4: OGD+oe-HGSNAT+Tun). Data are presented as mean ± SD (*n* = 3). *P < 0.05, **P < 0.01, ***P < 0.001, ****P < 0.0001.

To further elucidate whether HGSNAT mitigates OGD-induced oxidative stress by inhibiting ERS, we assessed oxidative stress levels and ERS-related signaling markers. Immunofluorescence staining showed that ROS fluorescence intensity was markedly elevated in the Tun-treated group compared with the HGSNAT-overexpressing condition (*P* < 0.0001, Figures 4A, B), indicating that ERS activation induced by Tun reverses the suppressive effect of HGSNAT overexpression on oxidative stress.

In line with the above results, Western blotting revealed that administration of Tun counteracted the inhibitory impact of HGSNAT overexpression on the ER stress pathway (Figure 4C). Specifically, relative to the HGSNAT-overexpressing group, the Tun-treated group exhibited markedly elevated protein abundance of GRP78 (*P* < 0.05, Figure 4D) and ATF4 (*P* < 0.05, Figure 4E), together with increased p-PERK/PERK (*P* < 0.01, Figure 4F) and p-eIF2α/eIF2α (*P* < 0.01, Figure 4G) ratios. This result indicates that ER stress induction led to reactivation of the PERK–eIF2α-ATF4 signaling cascade.

### 3.5 Specific activation of PERK by CCT020312 abrogates the HGSNAT-mediated protective phenotypes in OGD-injured HBMECs

**FIGURE 5:**
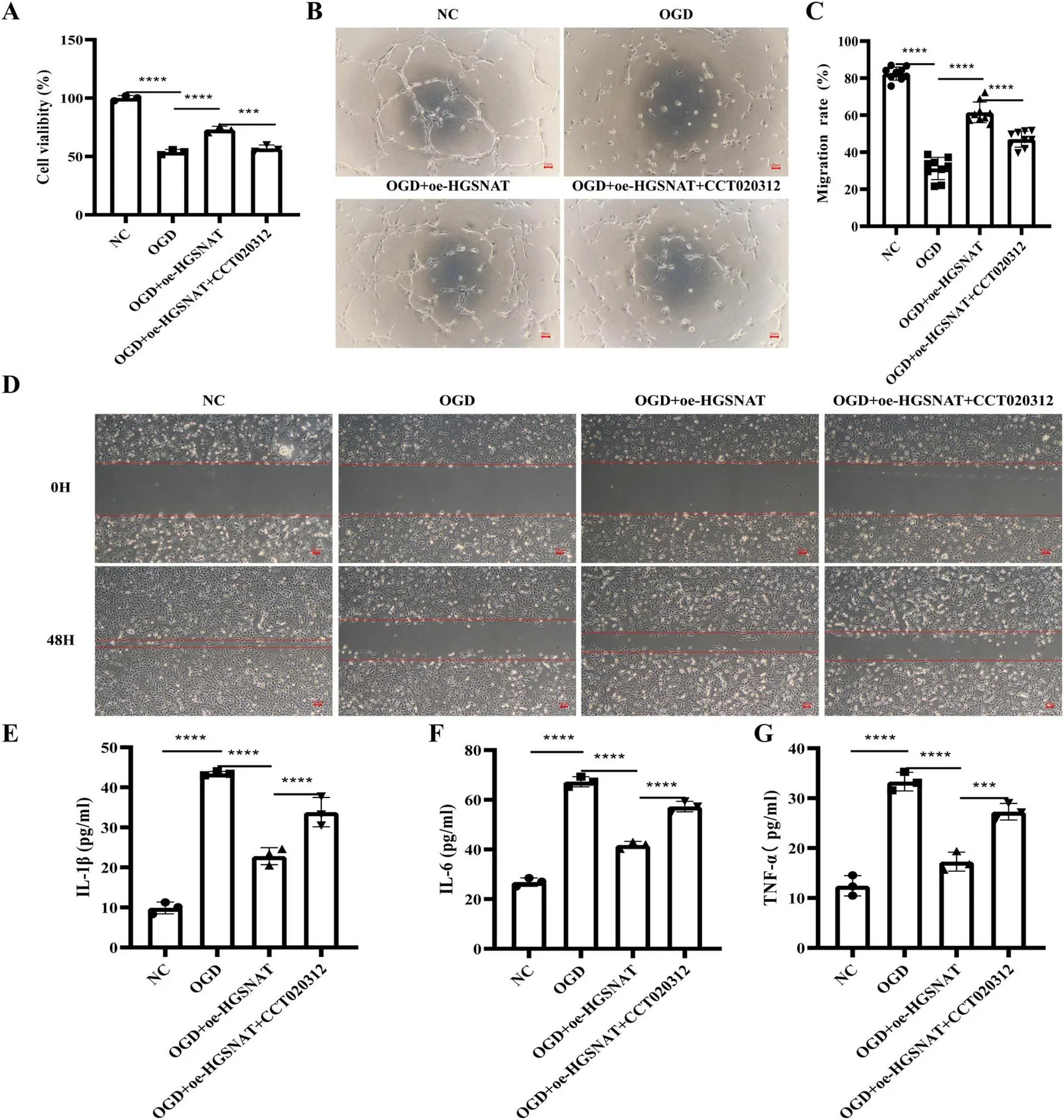
CCT020312-mediated activation of the PERK pathway reverses the protective effect of heparan-α-glucosaminide N-acetyltransferase (HGSNAT) on human brain microvascular endothelial cells (HBMEC) viability, angiogenesis, and migration under oxygen-glucose deprivation (OGD) challenge. **(A)** Cell survival fraction checked by CCK-8. **(B)** Capillary-like tube networks formed by HBMECs (scale bar = 50 μm). **(C,D)** Quantitative analysis and representative images of the scratch wound healing assay at 0 and 48 h (scale bar = 100 μm). **(E–G)** Extracellular release of inflammatory cytokines interleukin-1β (IL-1β), interleukin-6 (IL-6), and tumor necrosis factor-α (TNF-α) was examined by enzyme-linked immunosorbent assay (ELISA). Data are presented as mean ± SD (*n* = 3). ***P < 0.001, ****P < 0.0001.

While Tun acts as a general initiator of ER stress, we next sought to specifically validate the involvement of the PERK–eIF2α–ATF4 signaling pathway in HGSNAT-mediated endothelial protection. To achieve this, a highly selective PERK activator, CCT020312, was administered to OGD-treated HBMECs overexpressing HGSNAT. According to CCK-8 assay results, cell survival was significantly decreased in the CCT020312-treated group compared with the OGD+oe-HGSNAT group (*P* < 0.001, Figure 5A), indicating that specific PERK activation successfully counteracted the pro-survival benefits conferred by HGSNAT.

Furthermore, phenotypic analyses through tube formation assay (Figure 5B) demonstrated that CCT020312 treatment markedly impaired the angiogenic capacity enhanced by HGSNAT overexpression. Similarly, scratch wound healing assays (Figures 5C, D) revealed that specific activation of PERK significantly suppressed the migratory potential of HBMECs (*P* < 0.0001). In addition, ELISA results demonstrated that the levels of inflammatory mediators in cell culture supernatants, including IL-1β (*P* < 0.0001, Figure 5E), IL-6 (*P* < 0.0001, Figure 5F), and TNF-α (*P* < 0.001, Figure 5G), were remarkably elevated following CCT020312 administration compared with the OGD+oe-HGSNAT group. Together, these results reveal that specific activation of the PERK pathway reverses the protective and anti-inflammatory phenotypes mediated by HGSNAT overexpression.

### 3.6 CCT020312-mediated PERK activation reverses HGSNAT-suppressed oxidative stress and reactivates the PERK–eIF2α–ATF4 pathway

**FIGURE 6:**
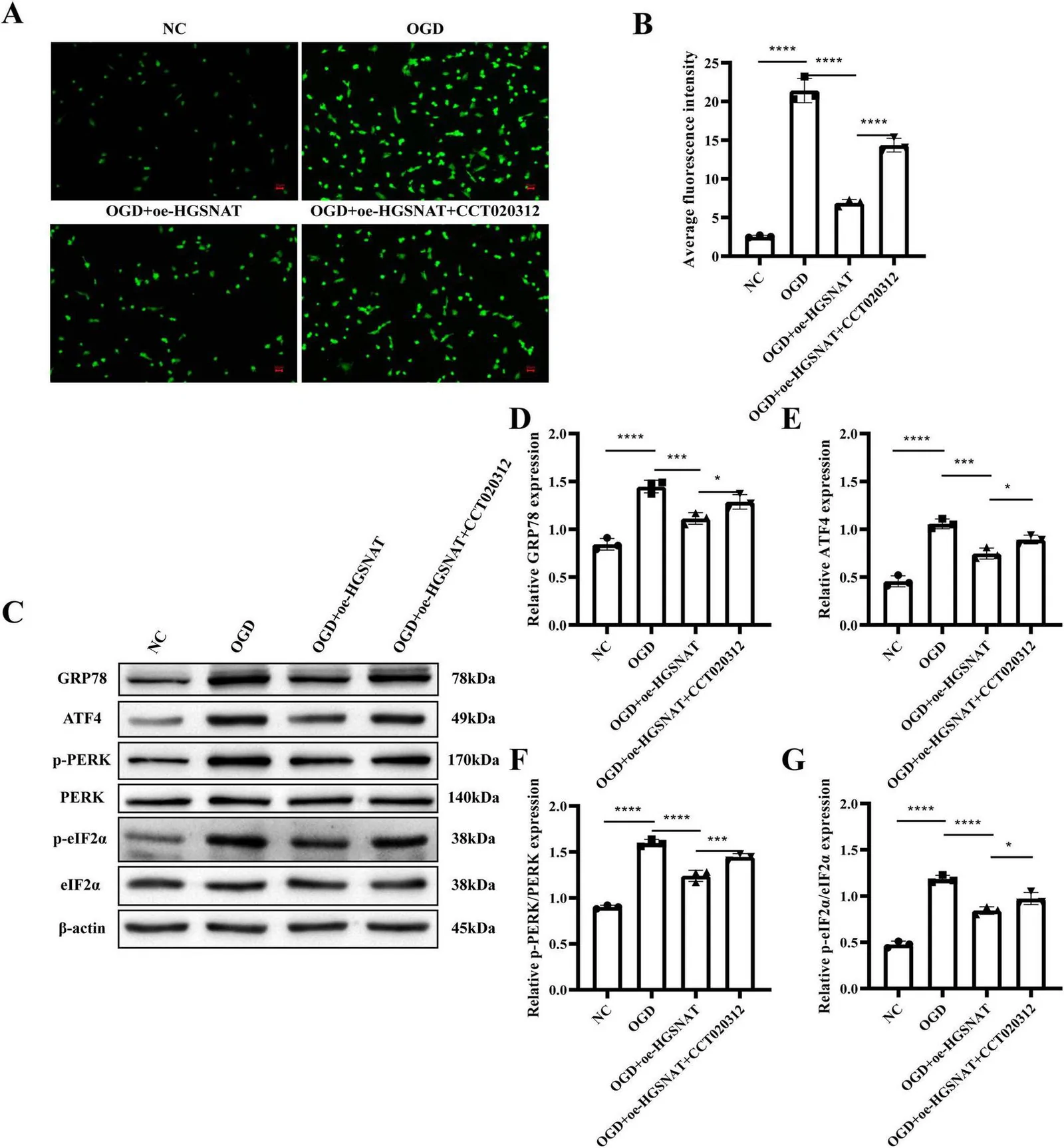
PERK activator CCT020312 reactivates reactive oxygen species (ROS) production and downstream ER stress markers suppressed by heparan-α-glucosaminide N-acetyltransferase (HGSNAT) overexpression. **(A,B)** 2’,7’-Dichlorodihydrofluorescein diacetate (DCFH-DA) cellular imaging and relative ROS fluorescence intensity quantification (scale bar = 50 μm). **(C)** Western blot signals for GRP78, ATF4, p-PERK, PERK, p-eIF2α, and eIF2α proteins. **(D–G)** Calculated protein expression changes for GRP78 and ATF4, along with phosphorylation ratios of p-PERK/PERK and p-eIF2α/eIF2α. Data are presented as mean ± SD (*n* = 3). *P < 0.05, ***P < 0.001, ****P < 0.0001.

To further substantiate the molecular mechanisms, we evaluated the effects of specific PERK activation on intracellular oxidative stress and ERS-related signaling markers. Immunofluorescence staining of ROS using DCFH-DA showed that ROS fluorescence intensity was significantly elevated in the CCT020312-treated group compared with the HGSNAT-overexpressing condition (*P* < 0.0001, Figures 6A, B), indicating that specific PERK activation reverses the suppressive effect of HGSNAT overexpression on oxidative stress.

Consistent with these findings, Western blot assays confirmed that CCT020312 administration successfully reactivated the PERK–eIF2α–ATF4 signaling cascade (Figure 6C). Specifically, relative to the OGD+oe-HGSNAT group, the CCT020312-treated group exhibited a significantly increased protein abundance of the ERS markers GRP78 (*P* < 0.05, Figure 6D) and ATF4 (*P* < 0.05, Figure 6E), along with remarkably elevated ratios of p-PERK/PERK (*P* < 0.001, Figure 6F) and p-eIF2α/eIF2α (*P* < 0.05, Figure 6G). These findings provide direct and robust evidence that HGSNAT alleviates endothelial injury and oxidative stress specifically via the inhibition of the PERK–eIF2α–ATF4 pathway.

### 3.7 HGSNAT upregulates GPX8 expression in HBMECs

GPX8 is a glutathione peroxidase localized to the ER and serves a critical function in modulating ER stress responses (Yang et al., 2025). To elucidate whether HGSNAT modulates ER stress via GPX8, we investigated the regulatory interplay between these two proteins.

**FIGURE 7:**
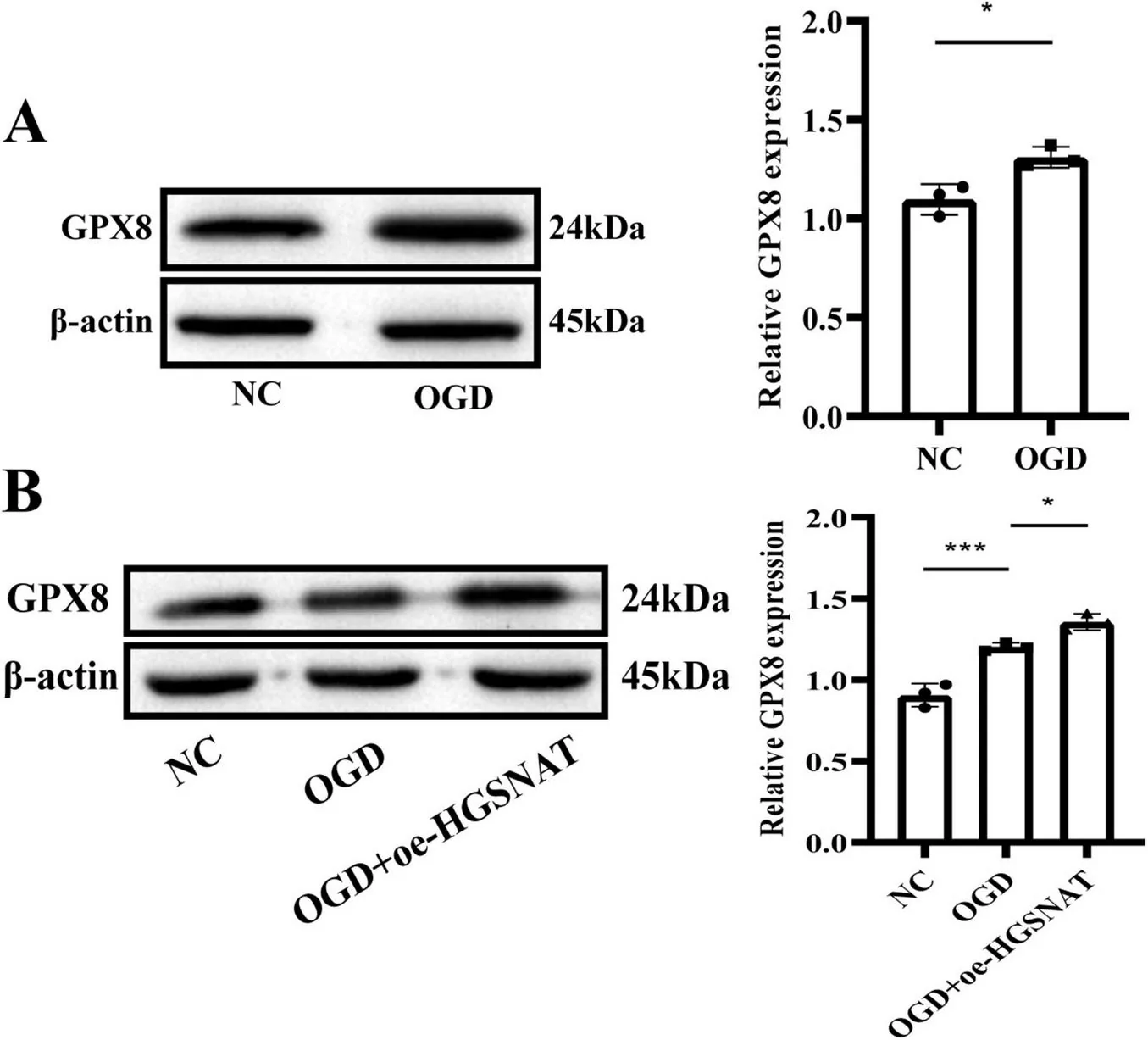
Heparan-α-glucosaminide N-acetyltransferase (HGSNAT) overexpression is associated with increased GPX8 levels in human brain microvascular endothelial cells (HBMECs) under OGD-stressed conditions. **(A)** Representative Western blot and relative expression summary of GPX8 in HBMECs under NC and oxygen-glucose deprivation (OGD) conditions. **(B)** Western blot tracks and relative protein levels of GPX8 expression in cells transfected with a control vector or HGSNAT overexpression plasmid under OGD insult. Data are presented as mean ± SD (*n* = 3). *P < 0.05, ***P < 0.001.

We first assessed the impact of OGD on GPX8 expression in HBMECs. GPX8 protein levels were markedly elevated in the OGD group versus control (*P* < 0.05, Figure 7A), suggesting that hypoxic-ischemic conditions trigger upregulation of GPX8. Then, HGSNAT was overexpressed in HBMECs under OGD-stressed conditions. GPX8 levels were markedly elevated in the HGSNAT-overexpressing group compared with the control (*P* < 0.05, Figure 7B). Interestingly, the observed upregulation of GPX8 after OGD—despite the concomitant downregulation of HGSNAT—suggests that these two molecules may respond to hypoxic-ischemic stress through distinct, independent pathways.

### 3.8 GPX8 knockdown abolishes HGSNAT-mediated protection against OGD-induced endothelial injury

To explore whether GPX8 contributes to the protective effects mediated by HGSNAT, we conducted functional experiments in which GPX8 was silenced in HGSNAT-overexpressing HBMECs subjected to OGD, and transfection efficiency was confirmed (Supplementary Figures 2C, D).

**FIGURE 8:**
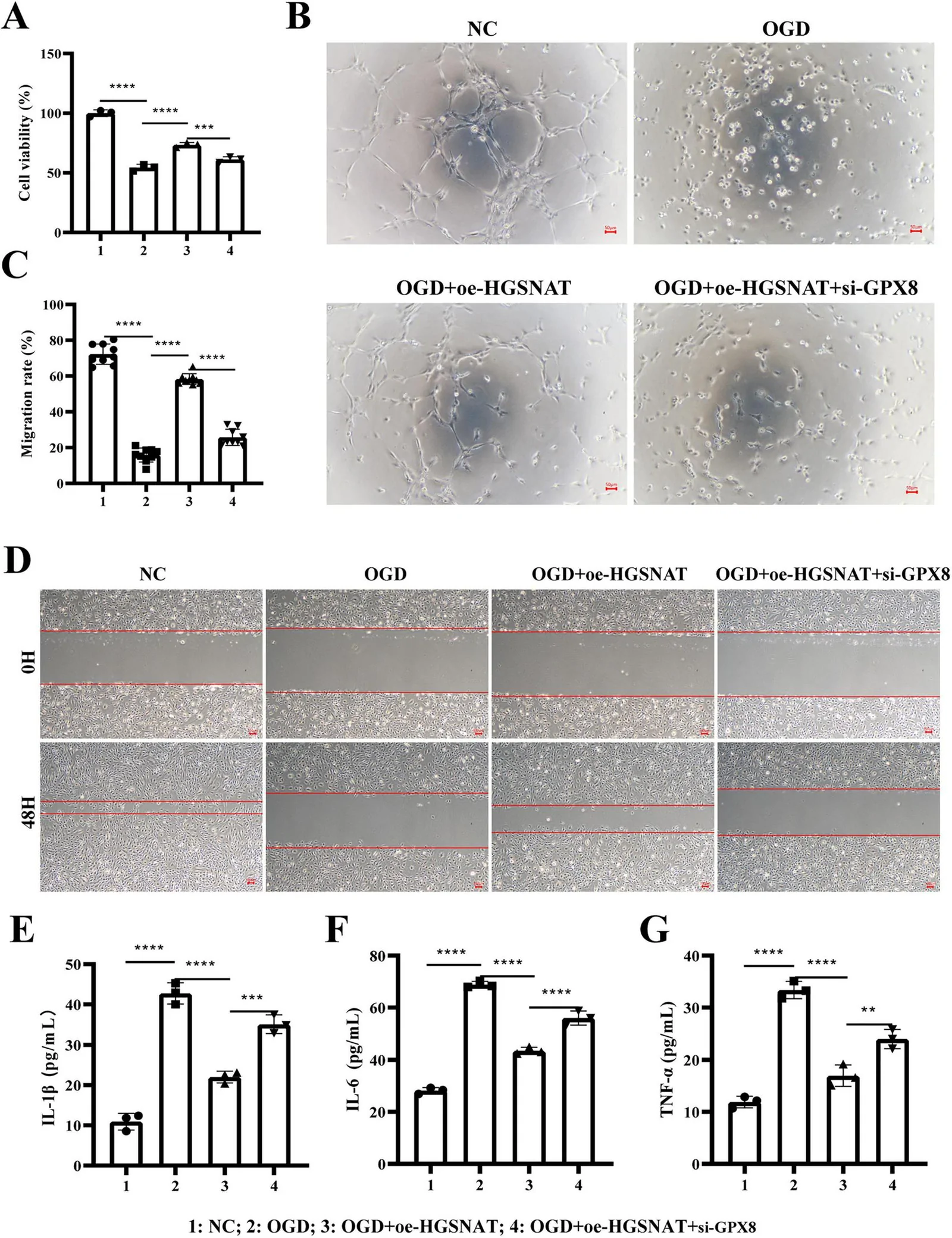
GPX8 knockdown blocks the protective and anti-inflammatory efficacy of heparan-α-glucosaminide N-acetyltransferase (HGSNAT) overexpression during oxygen-glucose deprivation (OGD)-induced endothelial damage. **(A)** CCK-8 measurement of HBMEC viability. **(B)** Evaluation of endothelial loop network integration by tube formation assay (scale bar = 50 μm). **(C,D)** Migration percentages and representative images of the scratch wound healing assay at 0 and 48 h (scale bar = 100 μm). **(E–G)** Inflammatory factor index [interleukin-1β (IL-1β), interleukin-6 (IL-6), and tumor necrosis factor-α (TNF-α)] in culture supernatant validated by enzyme-linked immunosorbent assay (ELISA). (Groups: 1: NC; 2: OGD; 3: OGD+oe-HGSNAT; 4: OGD+oe-HGSNAT+si-GPX8). Data are presented as mean ± SD (*n* = 3). **P < 0.01, ***P < 0.001, ****P < 0.0001.

CCK-8 assay results demonstrated that cell survival was markedly reduced in the GPX8-silenced group compared with the HGSNAT-overexpressing group (*P* < 0.001, Figure 8A), suggesting that knockdown of GPX8 impairs the cytoprotective effects mediated by HGSNAT overexpression. In addition, results from tube formation (Figure 8B) and scratch wound healing assays (Figures 8C, D) showed that silencing GPX8 significantly impaired the pro-angiogenic and pro-migratory effects induced by HGSNAT overexpression, reflected by a marked decrease in both tube-like structures and migrated cells (*P* < 0.0001, Figure 8C). In addition, ELISA results revealed that the abundance of inflammatory mediators—namely IL-1β (*P* < 0.001, Figure 8E), IL-6 (*P* < 0.0001, Figure 8F), and TNF-α (*P* < 0.01, Figure 8G)—was markedly elevated in the GPX8-silenced group relative to the HGSNAT-overexpressing group. Together, these results indicate that loss of GPX8 reactivates the inflammatory response triggered by OGD, thus significantly weakening the anti-inflammatory effects mediated by HGSNAT overexpression.

### 3.9 GPX8 silencing reverses HGSNAT-mediated inhibition of oxidative stress and the PERK-eIF2α-ATF4 pathway

**FIGURE 9:**
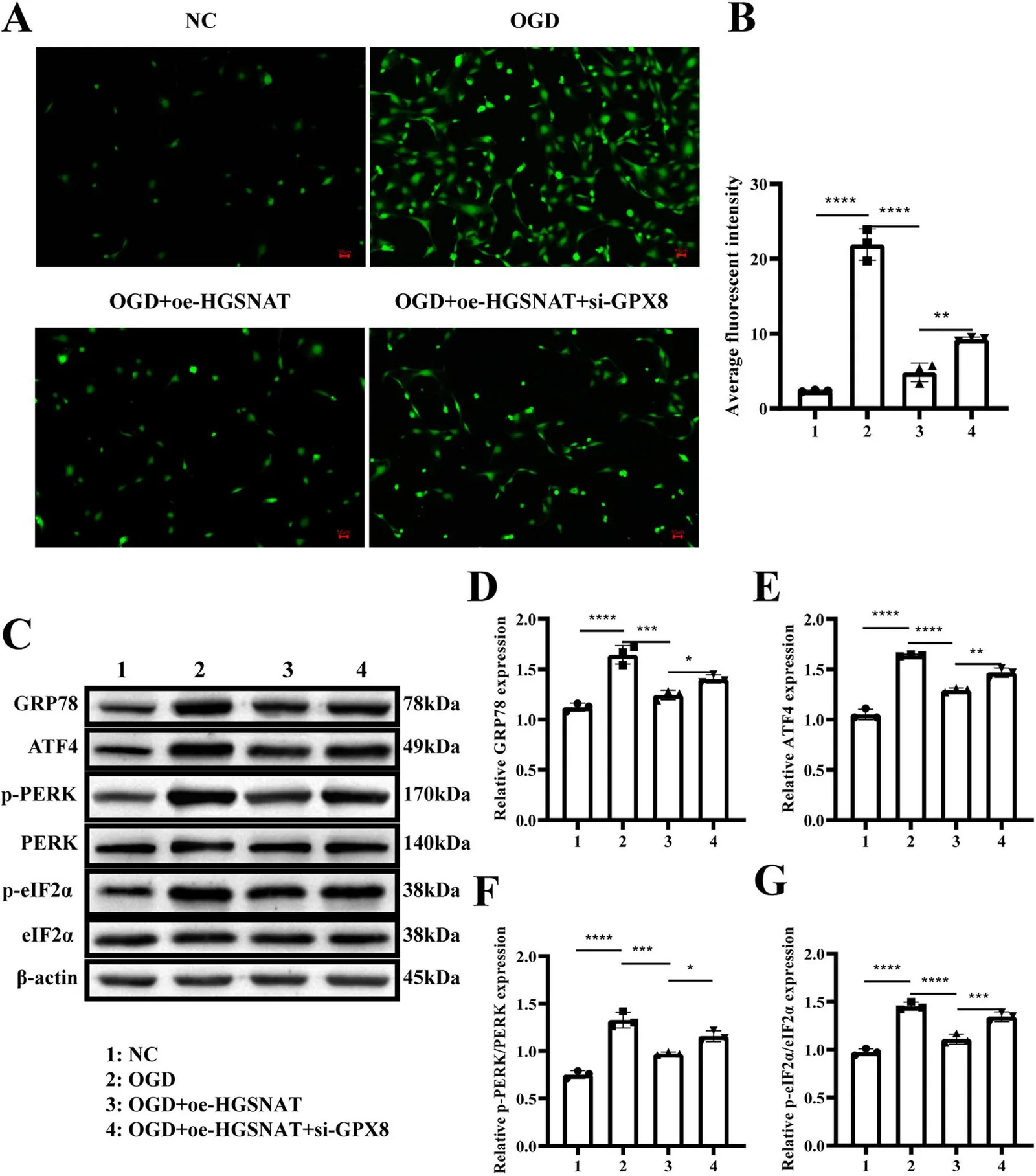
Silencing of GPX8 neutralizes the inhibitory effect of heparan-α-glucosaminide N-acetyltransferase (HGSNAT) on oxygen-glucose deprivation (OGD)-induced reactive oxygen species (ROS) synthesis and PERK-eIF2α-ATF4 axis activation. **(A,B)** Intracellular ROS profiles displayed by DCFH-DA green fluorescence and the associated statistical intensity (scale bar = 50 μm). **(C)** Blotted bands outlining GRP78, ATF4, p-PERK, PERK, p-eIF2α, and eIF2α protein levels. **(D–G)** Normalized gray value percentages for GRP78 and ATF4 expression, along with phosphorylation rates of P-PERK and P-eIF2α. (Groups: 1: NC; 2: OGD; 3: OGD+oe-HGSNAT; 4: OGD+oe-HGSNAT+si-GPX8). Data are presented as mean ± SD (*n* = 3). *P < 0.05, **P < 0.01, ***P < 0.001, ****P < 0.0001.

Reactive oxygen species fluorescence intensity was markedly elevated in the GPX8-silenced group versus the HGSNAT-overexpressing group (*P* < 0.01, Figures 9A, B), suggesting that knockdown of GPX8 reverses the suppressive effect of HGSNAT overexpression on oxidative stress.

Consistent with these observations, Western blot assays confirmed that GPX8 silencing substantially reversed the HGSNAT-mediated inhibition of the ERS pathway (Figure 9C). Specifically, the protein abundance of GRP78 (*P* < 0.05, Figure 9D) and ATF4 (*P* < 0.01, Figure 9E), and p-PERK/PERK (*P* < 0.05, Figure 9F) and p-eIF2α/eIF2α (*P* < 0.001, Figure 9G) ratios, were substantially elevated in the GPX8-silenced group versus the HGSNAT-overexpressing group. These findings indicate that GPX8 deficiency reactivates the PERK-eIF2α-ATF4 signaling pathway.

## 4 Discussion

Moyamoya disease is a progressive cerebrovascular disorder with distal internal carotid artery stenosis, occlusion, and aberrant collateral vessels (Shin et al., 2024). Endothelial dysfunction in brain microvascular cells constitutes a key pathological hallmark of MMD (Han et al., 2025; Ma et al., 2023). In this study, an OGD model was established using HBMECs to mimic the ischemic microenvironment associated with MMD in a controlled laboratory setting. The OGD model has been widely adopted in MMD research as an *in vitro* system that recapitulates the chronic cerebral hypoperfusion and ischemic stress characteristic of MMD pathophysiology. Specifically, MMD is characterized by progressive stenosis of the terminal internal carotid arteries, leading to a state of chronic hypoxia in the downstream brain microvasculature. Given that chronic cerebral hypoperfusion and resultant ischemic stress are cardinal features of MMD, the OGD model has been increasingly utilized to simulate the microvascular endothelial response to the MMD-specific ischemic microenvironment (He et al., 2026; Ihara et al., 2022; Shin et al., 2024). HBMECs, as the frontline cells sensing hemodynamic changes, undergo significant dysfunction under such conditions. Previous studies have demonstrated that OGD-induced injury in HBMECs effectively recapitulates the excessive oxidative stress and aberrant ERS observed in patient-derived vascular tissues (Ahmed et al., 2022; He et al., 2026; Lu et al., 2026). Furthermore, since the hallmark “moyamoya vessels” are a compensatory but fragile response to ischemia, studying the angiogenic and migratory capacities of HBMECs under OGD conditions provides critical insights into the fragile neoangiogenesis seen in MMD patients (Dorschel and Wanebo, 2023).

This model allows for systematic investigation of molecular pathways underlying endothelial dysfunction under conditions relevant to MMD-related ischemia. Using this established platform, we aimed to explore the functional involvement of HGSNAT in regulating endothelial function and to clarify its underlying molecular mechanisms. HGSNAT loss-of-function mutations cause mucopolysaccharidosis type IIIC (Musielak et al., 2024). Mutations in the HGSNAT gene that result in functional deficiency lead to mucopolysaccharidosis type IIIC, a condition marked by profound neurological deficits (Ludwig et al., 2023). The involvement of HGSNAT in cerebrovascular disorders, especially its potential contribution to cellular stress responses, remains unexplored. In this study, exposure to OGD led to a marked decrease in both the levels and enzymatic activity of HGSNAT in HBMECs. Given that the core pathological feature of MMD is prolonged cerebral hypoperfusion and ischemia, this finding suggests that HGSNAT may also be suppressed during the pathological progression of MMD. This phenomenon may represent an adaptive cellular response to hypoxic-ischemic stress (Wei et al., 2025); cells likely suppress non-essential metabolic processes, such as glycosaminoglycan degradation, to prioritize energy conservation and antioxidant defense (Wang et al., 2025). However, whether HGSNAT downregulation is directly involved in MMD-associated endothelial dysfunction requires further validation. Subsequent rescue experiments revealed that HGSNAT overexpression significantly ameliorated OGD-induced cytotoxicity, angiogenic deficits, and impaired migratory capacity. Notably, increasing HGSNAT expression reduced inflammatory mediator release, namely IL-1β, IL-6, and TNF-α, highlighting its protective function in preserving endothelial homeostasis under ischemic stress. These functional phenotypes are closely associated with the impaired angiogenic capacity and inflammatory microenvironment characteristic of MMD, suggesting that HGSNAT may be a key molecule in maintaining endothelial function in MMD.

Further investigation revealed that the reduction in HGSNAT mRNA levels occurred concomitantly with the upregulation of ERS markers, suggesting a potential pathological link between these events. Previous evidence has demonstrated that ERS is significantly upregulated in T cells from MMD patients, implicating ERS activation as a hallmark of MMD immunopathology (Liu et al., 2025). In conjunction with the concomitant downregulation of HGSNAT and upregulation of ERS markers observed in this study, this suggests that HGSNAT deficiency may be involved in mediating aberrant ERS activation in the vascular tissues of MMD. Various cellular stressors may disturb ERS, causing unfolded protein accumulation and subsequent unfolded protein response (UPR) induction (Pshezhetsky, 2015). As a central arm of the UPR, the PERK–eIF2α–ATF4 signaling axis reduces ER stress load by phosphorylating eIF2α, which suppresses global protein translation while selectively enhancing ATF4 expression to drive the transcription of antioxidant and pro-survival genes (Khan et al., 2026). Notably, missense mutations in HGSNAT cause enzyme misfolding, leading to its retention in the ER instead of proper trafficking to lysosomes, thereby linking HGSNAT dysfunction to the induction of ERS (Han et al., 2021). In line with these observations, the current study revealed that upregulation of HGSNAT alleviates ER stress by suppressing the PERK–eIF2α–ATF4 signaling axis, thereby exerting protective effects on endothelial cells and offering a potential mechanistic basis for targeting the aberrant ER stress observed in MMD.

The bidirectional interplay between ERS and oxidative stress has been extensively documented (Nunes and Demaurex, 2014). ERS exacerbates mitochondrial ROS generation by disrupting calcium homeostasis and redox balance (Victor et al., 2021). Conversely, oxidative stress compromises ER protein folding capacity, thereby intensifying ERS (Saqirile et al., 2024). This vicious cycle drives endothelial injury and vascular dysfunction (Ren et al., 2021), which is a central component of the vascular pathology progression in MMD. Consistent with this framework, we observed that HGSNAT upregulation significantly inhibited ROS accumulation in OGD-treated HBMECs. These findings suggest that HGSNAT exerts cytoprotective effects, at least in part, by interrupting the vicious cycle between ERS and oxidative stress, indicating its potential value in blocking the self-amplifying process of vascular injury in MMD.

The chronic hypoxic-ischemic microenvironment in MMD is increasingly recognized as an important driver of sterile inflammation. This concept may help reconcile the traditional view of non-inflammatory intimal thickening with the growing evidence of inflammatory activation observed in MMD. Our research shows that HBMECs exposed to OGD conditions exhibit significantly increased secretion of IL-1β, IL-6, and TNF-α, thereby recapitulating key features of the inflammatory microenvironment associated with ischemic injury (Abhinav et al., 2024; Dorschel and Wanebo, 2023; Liu et al., 2023). Mechanistically, the PERK–eIF2α–ATF4 signaling pathway not only mediates ER stress but also represents an important molecular link between ER stress and inflammatory signaling. Sustained activation of the UPR can activate NF-κB signaling and the NLR family pyrin domain-containing 3 (NLRP3) inflammasome, thereby leading to the maturation and secretion of IL-1β and other pro-inflammatory cytokines (Grootjans et al., 2016; Li et al., 2020). Moreover, ER stress-induced oxidative stress activates ROS-dependent redox-sensitive inflammatory pathways, further amplifying the inflammatory response (Grootjans et al., 2016; Victor et al., 2021). In our study, HGSNAT overexpression was associated with increased GPX8 expression and reduced inflammatory cytokine production. GPX8 knockdown attenuated these protective effects, suggesting that GPX8 contributes to HGSNAT-mediated suppression of ER stress and inflammation. These findings indicate that targeting the HGSNAT/GPX8-associated protective pathway may help alleviate chronic vascular inflammation and preserve cerebrovascular homeostasis during MMD progression (Dorschel and Wanebo, 2023).

Emerging evidence has implicated GPX8 as a crucial regulator of ERS adaptation (Yoshioka et al., 2022). As an ER-resident glutathione peroxidase, GPX8 maintains ER redox homeostasis by scavenging hydrogen peroxide generated during protein oxidative folding (Buday and Conrad, 2021). Furthermore, GPX8 localizes to mitochondria-associated ER membranes (MAMs), integrating ERS and redox signaling to regulate calcium homeostasis (Yoboue et al., 2017). Consistent with previous studies, OGD treatment increased GPX8 expression in HBMECs, supporting the concept that GPX8 acts as a stress-responsive cytoprotective protein. Previous studies have demonstrated that GPX8 expression can be induced by hypoxic stress through HIF-1α-dependent transcriptional regulation, suggesting that the increased GPX8 expression observed after OGD may represent an endogenous adaptive response aimed at maintaining ER redox balance and limiting oxidative damage under ischemic conditions (Bosello-Travain et al., 2015). Notably, the concurrent reduction of HGSNAT and elevation of GPX8 following OGD indicate that these two molecular changes may represent independent cellular responses to ischemic stress rather than a direct regulatory relationship. Interestingly, despite the opposite expression patterns of HGSNAT and GPX8 after OGD, GPX8 silencing markedly attenuated the protective effects of HGSNAT overexpression, indicating that GPX8 remains functionally required for HGSNAT-mediated cytoprotection. Rather than supporting a direct linear regulatory relationship, our findings suggest that HGSNAT and GPX8 may act cooperatively within a coordinated adaptive stress-response network that converges on the PERK–eIF2α–ATF4 pathway to preserve ER homeostasis during ischemic injury. This interpretation is also consistent with the emerging concept that endothelial adaptation to hypoxia is governed by multiple interacting cytoprotective mechanisms instead of a single signaling axis (Sato and Takeda, 2023; Yuan et al., 2024). Further studies are warranted to determine whether HGSNAT modulates GPX8 indirectly through alterations in cellular redox status or ER homeostasis, or whether both molecules are regulated by common upstream stress-responsive pathways.

The current study has several shortcomings. First, the findings are largely derived from *in vitro* experiments, and while several well-established animal models are available for future validation—including Rnf213-deficient mouse models that recapitulate MMD pathophysiology (Li et al., 2023; Morimoto et al., 2018), chronic cerebral hypoperfusion models (BCAS/BICAO) that mimic MMD-related ischemic microenvironments (Lim et al., 2024; Shibata et al., 2007; Watanabe et al., 1996), and transient middle cerebral artery occlusion (tMCAO) models for broader ischemic stroke relevance (Jiang et al., 2024; Longa et al., 1989)—the present study does not include *in vivo* data. Future investigations employing these complementary models could examine whether HGSNAT upregulation attenuates ER stress, reduces oxidative stress, preserves blood-brain barrier integrity, and improves functional outcomes, thereby validating the translational relevance of the HGSNAT-GPX8 axis. Second, although our functional experiments demonstrated that GPX8 contributes to the protective effects of HGSNAT against OGD-induced endothelial injury, the precise relationship between these two molecules remains to be elucidated. The opposite expression patterns of HGSNAT and GPX8 following OGD suggest that GPX8 upregulation may represent an adaptive stress response independent of HGSNAT under ischemic conditions. It is noteworthy that the increase in GPX8 expression following HGSNAT overexpression was observed under OGD stress rather than being a strong direct induction under basal conditions. This suggests that HGSNAT may support the ER protein-folding environment, thereby facilitating or synergizing with the adaptive upregulation of GPX8 to combat ischemic injury, rather than acting as its primary transcriptional activator. Whether HGSNAT influences GPX8 indirectly through modulation of ER homeostasis, cellular redox status, or other upstream stress-responsive pathways requires further investigation. Future studies integrating transcriptomic, proteomic, and mechanistic approaches will be valuable for clarifying the molecular interactions between HGSNAT and GPX8 during endothelial adaptation to ischemic stress.

## 5 Conclusion

This study reveals a protective role for HGSNAT against OGD-induced endothelial dysfunction, characterized by reduced inflammation and oxidative stress. HGSNAT and GPX8 appear to function cooperatively in suppressing the PERK–eIF2α–ATF4 ERS pathway, thereby mitigating endothelial injury. GPX8 knockdown attenuated the protective effects of HGSNAT, supporting the involvement of GPX8 in HGSNAT-mediated endothelial protection. Collectively, these findings deepen our understanding of the molecular mechanisms underlying MMD and highlight HGSNAT and GPX8 as potential therapeutic targets.

## Data Availability

The original contributions presented in this study are included in the article/Supplementary material, further inquiries can be directed to the corresponding author.

## Supplementary material

Supplementary Figure 1 Time-course effects of oxygen-glucose deprivation (OGD) treatment on cell viability, heparan-α-glucosaminide N-acetyltransferase (HGSNAT) expression/activity, and GPX8 expression in human brain microvascular endothelial cells (HBMECs). **(A)** Time-dependent changes in HBMEC viability following OGD exposure for 0 (NC), 3, 6, 9, and 12 h, assessed by CCK-8 assay. **(B,C)** Relative mRNA expression levels **(B)** and enzymatic activity **(C)** of HGSNAT in HBMECs after different durations of OGD treatment. **(D)** Representative Western blot bands showing GPX8 protein expression levels across the OGD time course, with β-actin as the internal loading control. **(E)** Statistical quantification of relative GPX8 protein expression levels normalized to β-actin. Data are presented as mean ± SD from three independent experiments (*n* = 3). ***P* < 0.01, *****P* < 0.0001 versus NC group.

Supplementary Figure 2 Validation of cell transfection efficiencies for heparan-α-glucosaminide N-acetyltransferase (HGSNAT) overexpression and GPX8 knockdown in human brain microvascular endothelial cells (HBMECs) under control and oxygen-glucose deprivation (OGD) conditions. **(A,B)** Verification of HGSNAT overexpression efficiency. HGSNAT enzymatic activity **(A)** and relative HGSNAT mRNA expression levels **(B)** were measured in cells assigned to NC, OGD, OGD+oe-NC, and OGD+oe-HGSNAT groups. **(C)** Representative Western blot tracks evaluating the knockdown efficacy of si-GPX8, with β-actin as the internal loading control. **(D)** Statistical quantification of relative GPX8 protein expression levels across NC, OGD, OGD+si-NC, and OGD+si-GPX8 groups. Data are presented as mean ± SD from three independent experiments (*n* = 3). ***P* < 0.01, ****P* < 0.001, *****P* < 0.0001 versus the corresponding control group.
